# AIB1 is a novel target of the high‐risk HPV E6 protein and a biomarker of cervical cancer progression

**DOI:** 10.1002/jmv.27795

**Published:** 2022-04-27

**Authors:** Jonathan Miller, Aleksandra Dakic, Megan Spurgeon, Francisco Saenz, Bhaskar Kallakury, Bo Zhao, Junran Zhang, Jian Zhu, Qin Ma, Ying Xu, Paul Lambert, Richard Schlegel, Anna T. Riegel, Xuefeng Liu

**Affiliations:** ^1^ Department of Pathology, Center for Cell Reprogramming Georgetown University Medical School Washington District of Columbia USA; ^2^ McArdle Laboratory for Cancer Research, Department of Oncology University of Wisconsin‐Madison School of Medicine and Public Health Madison Wisconsin USA; ^3^ Department of Oncology, Lombardi Comprehensive Cancer Center Georgetown University Medical School Washington District of Columbia USA; ^4^ Department of Medicine, Brigham and Women's Hospital Harvard Medical School Boston Massachusetts USA; ^5^ Department of Radiation Oncology, Wexner Medical Center The Ohio State University Columbus Ohio USA; ^6^ The James Comprehensive Cancer Center The Ohio State University Columbus Ohio USA; ^7^ Department of Pathology, Wexner Medical Center The Ohio State University Columbus Ohio USA; ^8^ Department of Biomedical Informatics, College of Medicine The Ohio State University Columbus Ohio USA; ^9^ Computational Systems Biology Lab, Department of Biochemistry and Molecular Biology and Institute of Bioinformatics The University of Georgia Athens Georgia USA

**Keywords:** AIB1, cervical cancer, E6, E7, oncoproteins, P53, papillomavirus

## Abstract

The high‐risk human papillomaviruses (HPV‐16, ‐18) are critical etiologic agents in human malignancy, most importantly in cervical cancer. These oncogenic viruses encode the E6 and E7 proteins that are uniformly retained and expressed in cervical cancers and required for maintenance of the tumorigenic phenotype. The E6 and E7 proteins were first identified as targeting the p53 and pRB tumor suppressor pathways, respectively, in host cells, thereby leading to disruption of cell cycle controls. In addition to p53 degradation, a number of other functions and critical targets for E6 have been described, including telomerase, Myc, PDZ‐containing proteins, Akt, Wnt, mTORC1, as well as others. In this study, we identified Amplified in Breast Cancer 1 (AIB1) as a new E6 target. We first found that E6 and hTERT altered similar profiling of gene expression in human foreskin keratinocytes (HFK), independent of telomerase activity. Importantly, AIB1 was a common transcriptional target of both E6 and hTERT. We then verified that high‐risk E6 but not low‐risk E6 expression led to increases in AIB1 transcript levels by real‐time RT‐PCR, suggesting that AIB1 upregulation may play an important role in cancer development. Western blots demonstrated that AIB1 expression increased in HPV‐16 E6 and E7 expressing (E6E7) immortalized foreskin and cervical keratinocytes, and in three of four common cervical cancer cell lines as well. Then, we evaluated the expression of AIB1 in human cervical lesions and invasive carcinoma using immunohistochemical staining. Strikingly, AIB1 showed positivity in the nucleus of cells in the immediate suprabasal epithelium, while nuclei of the basal epithelium were negative, as evident in the Cervical Intraepithelial Neoplasia 1 (CIN1) samples. As the pathological grading of cervical lesions increased from CIN1, CIN2, CIN3 carcinoma in situ and invasive carcinoma, AIB1 staining increased progressively, suggesting that AIB1 may serve as a novel histological biomarker for cervical cancer development. For cases of invasive cervical carcinoma, AIB1 staining was specific to cancerous lesions. Increased expression of AIB1 was also observed in transgenic mouse cervical neoplasia and cancer models induced by E6E7 and estrogen. Knockdown of AIB1 expression in E6E7 immortalized human cervical cells significantly abolished cell proliferation. Taken together, these data support AIB1 as a novel target of HPV E6 and a biomarker of cervical cancer progression.

## 1 INTRODUCTION

Cervical cancer is the most common gynecologic cancer and the leading cause of cancer‐related deaths in women worldwide.^1^, ^2^ Approximately 527 000 women are diagnosed with cervical cancer and >260 000 women die of this disease each year, equal to almost one‐tenth of global cancer deaths in women. The National Cancer Institute estimates that there are ~290 000 women living with cervical cancer in the United States.^3^ Nearly all cases of cervical carcinoma are associated with infection by human papillomaviruses (HPV),^4^, ^5^, ^6^, ^7^, ^8^, ^9^ suggesting the role of HPV in cervical cancer development. While the implementation of HPV vaccination has had a major impact on reducing persistent genital HPV infections,^10^ a large proportion of women (approximately 40%) in the United States remain unvaccinated. Worldwide the HPV vaccination rate varies but in developing regions such as Africa and Asia, approximately 99% of the girls between the ages of 10−20 years remain unvaccinated.^11^ Currently, prophylactic HPV vaccines will not eliminate the virus from infected individuals. There are many aged people who have been infected before HPV vaccines were successfully developed. Thus, HPV‐associated human cancers will remain a significant challenge in the next 20−40 years.^1^, ^10^, ^12^, ^13^ Compounded with the inability of these countries to afford the cost of widespread vaccination programs, it is clear that alternative approaches to treating cervical cancer and precancer will be needed for the foreseeable future. This is especially important since the survival rates of recurrent cervical cancer and advanced‐stage cancer are very low and are considered to be incurable. Effective therapeutic and early‐intervention options for cervical cancer are not available because a detailed mechanism underlying carcinogenic progression is unclear. High‐risk HPV DNA, which encodes the E6 and E7 oncogenes, is present in nearly all cervical cancers and HPV is thereby considered a necessary agent for cancer initiation and development.^9^ Although epidemiological studies show that the estimated lifetime risk of HPV infection in women is more than 75%, the majority of infected women never develop cancer. Therefore, HPV alone is insufficient for cancer development. Unknown factors unique to individual hosts appear to contribute to dysplastic transformation and progression. In addition, the genetic analysis of cervical cancer indicates that there are no commonly occurring mutations that give insight into the initiation or progression of the malignancy.^2^ However, it has been shown that telomerase activity increases with advancing cervical dysplasia and that TERC (telomerase RNA component), one of the components of the telomerase complex, is overexpressed (mainly by amplification) in nearly all human cervical cancers (>90%).^14^, ^15^ Two studies demonstrate that TERC is amplified in 20%−21% of mild dysplasia of the cervix (CIN I), 50%−68% of moderate dysplasia (CIN II), 81%−82% of severe dysplasia (CIN III), and 95%−100% in invasive cancer.^16^, ^17^ TERT, the second component of the telomerase complex, is also increasingly expressed in the cascade of cervical dysplasia.^18^ Therefore, identifying the common factors regulated by E6 and TERC might be an important approach to understanding the etiology of HPV‐associated cervical cancer and provide a potential target for cancer therapy.

More than 600 genomes of individual papillomavirus types have been sequenced and characterized.^19^ The HPV life cycle is highly regulated in the host stratified cutaneous and mucosal epithelia.^19^, ^20^, ^21^ E5, E6, and E7 proteins from HR HPVs are required to evade innate immune responses of the host cells and to create an environment to support HPV replication in host cells.^19^, ^22^, ^23^, ^24^, ^25^ A long‐term consequence of these HPV/host interactions, especially proliferation manipulated by these interactions becomes a high risk for the transformation of the host cells which is not a primary intent of HPV infection. E6 and E7 proteins from HR‐HPVs are essential for the immortalization of keratinocytes and the progression to transformation.^26^, ^27^ The two oncogenes are expressed in both cervical cancer cell lines and primary and metastatic cervical cancers.^28^, ^29^, ^30^ Studies using RNA interference directed against these two genes are sufficient to senesce cervical cancer cells.^31^ E7 acts at the restriction point to bind the cellular Retinoblastoma (RB) protein, disassociating RB from E2F, leading to transcription of S phase genes and uncontrolled cell growth.^27^, ^32^, ^33^, ^34^ E6 complexes with the cellular E6‐associated protein (E6AP), functioning as an E3 ubiquitin ligase in p53 ubiquitination, thereby tagging p53 for degradation by the 26S proteasomal degradation pathway.^35^, ^36^, ^37^, ^38^, ^39^ However, E6 mutants defective for p53 degradation can still immortalize cells suggesting E6 functions independently of p53 in immortalization.^40^, ^41^, ^42^, ^43^ In addition to p53 degradation, other functions and targets of E6 have been described.^44^, ^45^, ^46^ For example, E6 induces telomerase activity in epithelial cells,^47^, ^48^ a function of E6 that also requires interaction with E6AP.^49^, ^50^, ^51^, ^52^ E6 has also been shown to activate Akt, Wnt, mTORC1, and other well‐known cancer signaling pathways.^45^, ^53^, ^54^, ^55^, ^56^, ^57^, ^58^, ^59^, ^60^ Several studies indicate the role of HPV in histone modification and chromatin remodeling. For example, E7 interacts with the chromatin silencing polycomb group complexes via E2F6 interaction^61^ as well as induces histone demethylase expression.^62^ E6 inhibits the histone acetyltransferase (HAT) activity of the important coactivator p300.^63^, ^64^, ^65^ Hsu et al.^66^ showed that E6 also targets histone methyltransferases, including CARM1, to modulate gene transcription. Amplified in Breast Cancer 1 (AIB1)/SRC‐3/NCOA3/ACTR is a member of the p160 nuclear receptor coactivator family. It promotes the transcriptional activity of multiple nuclear receptors and transcription factors.^67^, ^68^ As the name indicates, AIB1 is amplified in 2%−10% of breast cancer samples^69^, ^70^, ^71^ but increased *AIB1* mRNA levels are found in as high as 64% of human breast tumors.^72^, ^73^ Additionally, we and others have shown direct interaction with E6AP and AIB1.^74^, ^75^ Like AIB1, E6AP has been shown to have a role in steroid receptor coactivation and may work in concert with AIB1 for this function.^76^ Recent studies from Riegel's laboratory and others indicated a role of AIB1 isoform in the invasion and progression of breast cancer.^74^, ^77^, ^78^, ^79^, ^80^, ^81^, ^82^

While AIB1 plays a clear role in nuclear receptor biology, it has also been implicated in chromatin remodeling. AIB1 was isolated as the p300/CBP cointegrator‐associated protein (p/CIP) based on its ability to interact with the C terminus of CBP and has been shown to bind to p300 as well.^68^, ^83^ Interestingly, AIB1 has been shown to have weak HAT activity of its own.^68^ This evidence places AIB1 in complex with many of the proteins regulated by E6. The activity and stability of AIB1 are regulated via methylation by the E6‐targeted CARM1 methyltransferase.^66^, ^84^ Here, we explore the role of AIB1 in HPV‐associated carcinogenesis. We identified AIB1 as a novel target of HPV E6, demonstrating that E6 induces increased expression of AIB1 mRNA and protein in the human foreskin and cervical keratinocytes as well as E6E7 immortalized cells. We also found increased levels of AIB1 protein in cervical cancer lines. Our in vivo data of IHC staining of human CIN and invasive carcinoma, and mouse cervical cancer models demonstrated that increases in AIB1 correlated with clinical staging, suggesting a potential biomarker for cervical cancer progression. Finally, knockdown of AIB1 expression abolished the proliferation of HPV E6E7immortalized cervical keratinocytes.

## 2 MATERIALS AND METHODS

### 2.1 Plasmids and retroviruses

The following plasmids and corresponding retroviruses were described previously^40^, ^50^: pLXSN vector and pJS55 vector, pLXSN‐16E6, pLXSN‐6bE6, pLXSN‐16E7, pLXSN‐16E6E7, pLXSN‐E6Δ123‐127, pLXSN‐E6mSAT, pLXSN‐E6ΔPDZ, pJS55‐16E6, pJS55‐6bE6, pJS55‐16E7, pJS55‐16E6E7, pJS55‐E6Δ123‐127, pJS55‐E6mSAT, pJS55‐E6ΔPDZ.

### 2.2 Cell culture and generation of stable cell lines

Primary human foreskin keratinocytes (HFKs) were isolated and cultured from neonatal foreskins as described.^58^. Primary human ectocervical keratinocytes (HECs) were derived from fresh cervical tissue similarly and obtained after hysterectomy for benign uterine diseases.^85^ Standard trypsinization procedures were used to isolate the keratinocytes, which were cultured in serum‐free keratinocyte medium supplemented with 50 μg/ml of bovine pituitary extract and 25 ng/ml of recombinant epidermal growth factor (Invitrogen). The cells were cultured in serum‐free keratinocyte growth media (Invitrogen) supplemented with gentamycin (50 µg/ml). Primary HFKs and HECs were transduced with amphotropic pLXSN retroviruses expressing HPV‐16E6 and HPV16‐E7, or E6 wt or its mutants (see above). Retrovirus‐transduced cells were selected in G418 (100 µg/ml) for 5 days. Resistant colonies were pooled and passaged every 3−4 days (1:4 ratio for HFKs and HECs). HeLa, C33A, SiHa, and Caski cells were maintained in a complete dulbecco's modified Eagle medium. All cells were cultured on plastic tissue culture dishes or flasks.

### 2.3 Tissue

Patient samples were acquired through the Histopathology and Tissue Shared Resource at the Lombardi Comprehensive Cancer Center (Washington, DC). Twenty‐one cervical tissues were acquired which represented different pathological stages—one normal tissue core and five tissue cores for each of the following pathological stages: Cervical Intraepithelial Neoplasia Stage 1 (CIN1), CIN2, CIN3 or carcinoma in situ, and invasive cervical carcinoma.

### 2.4 cDNA and quantitative real‐time PCR

SuperScript III Reverse Transcriptase kit (Invitrogen) was used to perform reverse transcription PCR (RT‐PCR), as previously described.^36^ Reactions were annealed and analyzed using a Bio‐Rad iCycler and accompanying software (Bio‐Rad Laboratories). Primer sets used include the following: AIB1‐F: 5′ GTG GCT CTA TTC CCA CAT TG 3′; AIB1‐R: 5′ CCA GTT GGT TAG ATG CTG CT 3′; GAPDH‐F: 5′ TCTCCTCTGACTTCAACAGC 3′; GAPDH‐R: 5′ GAAATGAGCTTGACAAAGTG 3′.

### 2.5 Western blot

Standard Western blot analysis was carried out using whole‐cell protein lysates and primary antibodies against AIB1 (1:1000, 5E11; Cell Signaling), P53 (1:1000, Pab 1801; Santa Cruz), and HPV16‐E7 (1:1000, ED17; Santa Cruz); and a secondary antibody with HRP conjugation and detected by chemiluminescence (anti‐rabbit IgG or anti‐mouse IgG; Santa Cruz Biotechnology). 10−20 µg of lysate protein was loaded in each well. Western blot analysis was performed as described previously.^77^ Equal protein sample loading was monitored using an anti‐ACTIN (1:5000; Sigma) or anti‐GAPDH (1:2000, FL‐335; Santa Cruz).

### 2.6 Immunohistochemistry

Immunohistochemistry of cervical tissue was performed for AIB1. Five micron sections from formalin‐fixed, paraffin embedded tissues were de‐paraffinized with xylenes and rehydrated through a graded alcohol series. Heat‐induced epitope retrieval was performed by immersing the tissue sections at 98°C for 20 min in 10 mM citrate buffer (pH 6.0) with 0.05% Tween. Immunohistochemical staining was performed using the VectaStain Kit from Vector Labs according to the manufacturer's instructions. Briefly, slides were treated with 3% hydrogen peroxide for 10 min. Endogenous biotin was blocked using an avidin/biotin blocking kit from Invitrogen. The slides were then treated with 10% normal goat serum and exposed to primary antibodies AIB1 (1:250; Cell Signaling) for 1 h at room temperature. Slides were exposed to appropriate biotin‐conjugated secondary antibodies (Vector Labs), Vectastain ABC reagent, and DAB chromagen (Dako). Slides were counterstained with hematoxylin (Harris Modified Hematoxylin; Fisher) at a 1:17 dilution for 2 min at RT, blued in 1% ammonium hydroxide for 1 min at room temperature, dehydrated, and mounted with Acrymount. Consecutive sections with the omitted primary antibody were used as negative controls.

### 2.7 Luciferase reporter assay

HFKs were transiently cotransfected using Lipofectamine 2000 reagent (Invitrogen) according to the protocol provided by the manufacturer. Cotransfections were performed using 1.2 μg of an AIB1 reporter plasmid (pGL3‐ACTR‐1.6 kb from Dr. Hong‐Wu Chen^86^) and 500 ng of each expression vector as indicated (pJS55‐HPV‐16 E6 wt or mutants Δ123‐127, SAT, or ΔPDZ or empty vector for basal promoter activity). Cells were also cotransfected with 2 ng of the pRL‐CMV plasmid (Promega), which contains the *Renilla reniformis* luciferase gene as a transfection control. Firefly and Renilla luciferase activities were measured 24 h after transfection using the dual‐luciferase reporter assay system (Promega) and Veritas microplate luminometer (Turner Biosystems).

### 2.8 Short hairpin RNA constructs and lentivirus infection

The AIB1 gene was targeted as previously described.^77^ Briefly, a density of 5 × 10^5^ cells were seeded 24 h before lentiviral infection with either control scramble shRNA, or AIB1 shRNA #1 targeting exon 6 (5′‐TGGTGAATCGAGACGGAAACA‐3′), and #2 targeting exon 14 (5′‐GCAGTCTATTCGTCCTCCATA‐3′). Lentiviral particles were added to cells with packaging plasmid (pCMV‐dR8.2dvpr) and envelope plasmid (pCMV‐VSVG) purchased from Addgene.

### 2.9 Cell proliferation assay

Five thousand control or AIB1 shRNA infected cells were seeded in 16‐well E‐Plate VIEW (ACEA #AE06324738001) arrays and growth rate/proliferation was recorded as cell index by real‐time monitoring using an electric cell‐substrate impedance sensor system (ECIS) over 4 days.

### 2.10 Cell migration assay

20−100 × 10^5^ control or AIB1 shRNA infected cells were seeded in the top chamber of 16‐well CIM‐Plate (ACEA #05665817001) in serum‐free media. Migration from the top chamber to the bottom chamber across a permeable membrane was recorded as cell index by real‐time monitoring using ECIS over 48 h. Media containing 0%, 5% or 10% serum was added to the bottom chamber and used as a chemokine attractant.

## 3 RESULTS

### 3.1 AIB1 expression is increased in HFKs with HPV16 E6 and hTERT independent of hTERT catalytic function

**Figure 1:**
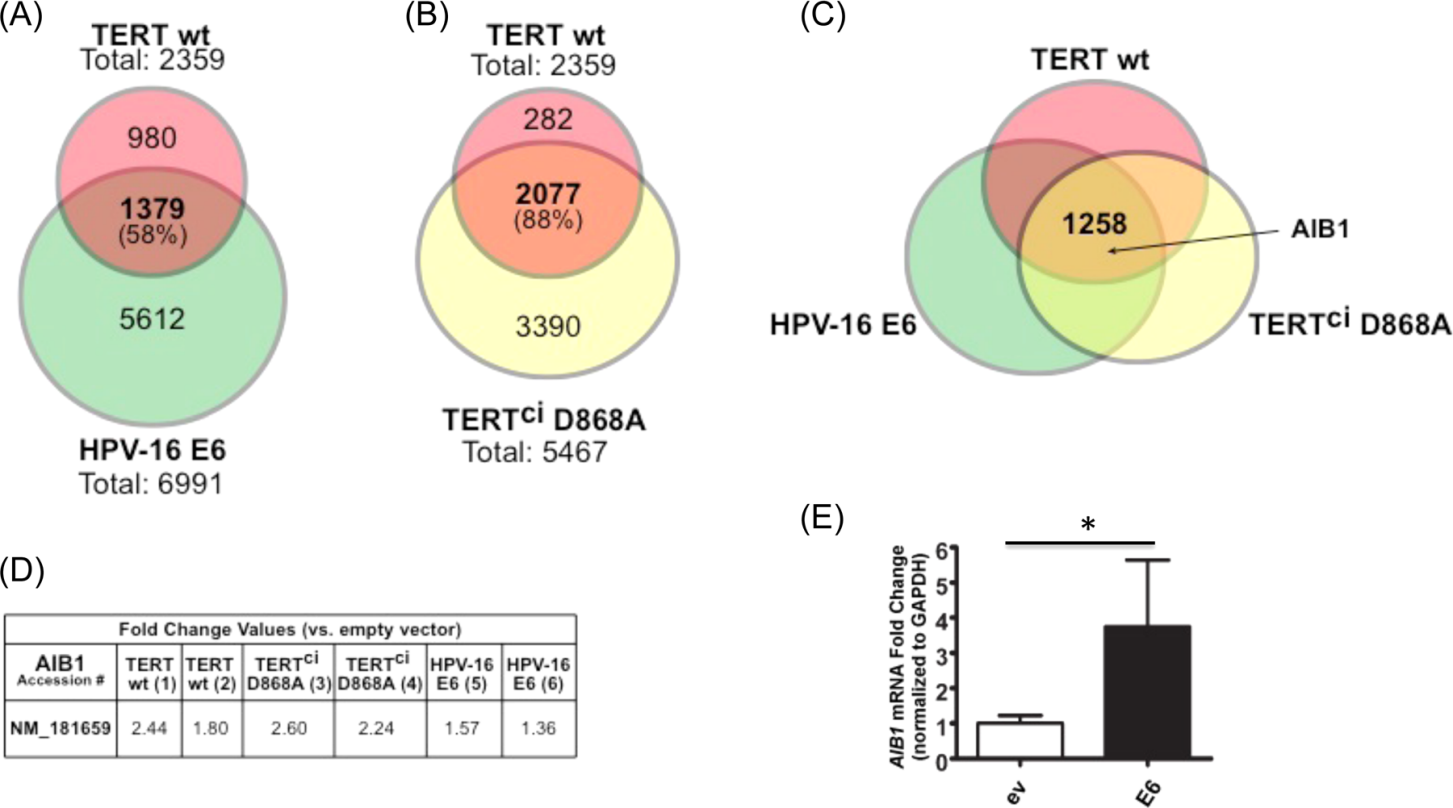
HPV16 E6 and hTERT alter the expression of overlapping gene sets and induce AIB1 expression independent of enzymatic activity. Primary HFKs were stably transduced with either E6, wild‐type hTERT, mutant hTERT (D868A), or control vector. Samples were submitted for whole‐genome expression array analysis (Figure S1). Expression profile changes shown by wild‐type hTERT are compared to expression profile changes in E6 (A) and hTERT mutant (B). As significant amount of the expression changes seen in E6 (6991 total changes) were also altered by wild‐type hTERT (1379, representing 20% of the E6 changes). Conversely, more than half of the hTERT changes (58%, 1379 of 2359 changes) were also seen by E6. Of the 2359 genes altered by wild‐type hTERT, 2077 of them (88%) were also altered by the hTERT mutant. (C) 1258 changes were shared by E6, wild‐type hTERT, and hTERT mutant, including AIB1. (D) Numerical fold change values are shown for all arrays as they correspond to two probes. NM_181659, this accession numbers represent AIB1 mRNA sequence. Quantitative RT‐PCR was performed on the E6 and hTERT or hTERT mutant (E) with gene‐specific primers for AIB1 to validate the array results, normalized to GAPDH. *n* = 3. Bars represent mean ± SD. *p < 0.05.

Our previous studies have shown that HPV E6 activates hTERT promoter and interacts with hTERT, thereby increasing telomerase in human host cells. We have also shown the extratelomeric functions of hTERT during immortalization of human keratinocytes by HPV E6E7.^41^, ^42^, ^43^ hTERT has been previously described to alter gene expression in various model systems. To study hTERT expression profile changes in the background of primary epithelial cells, we stably expressed E6, wild‐type hTERT (hTERT wt), or hTERTci D868A (a catalytically inactive protein) in primary HFKs and conducted an array‐based whole‐genome expression analysis (Figure S1A). Dye swap comparisons were performed with the arrays (Figure S1B). In all array pairs, the dye swap comparisons showed greater than 75% of the changes to be directionally consistent across the paired arrays. These served as an internal validation of the hybridization. Because E6 is a known activator of the hTERT protein, expression changes shared by hTERT and E6 could represent hTERT‐dependent E6 targets. As expected, a significant amount of mRNA expression changes seen in E6 was also altered by hTERT wt (1379 of 6991, or 20% of E6 changes with fold change >1.33 and *p*‐value < 0.01) (Figure 1A). More than half of the hTERT changes (58%, 1379/2359) were also seen by E6, suggesting that changes seen in hTERT‐expressing cells are also altered by E6‐ expressing cells, possibly through an hTERT‐dependent pathway. To pursue whether these expression changes seen in hTERT wt were dependent on changes in telomere biology, we also expressed a catalytically inactive mutated protein hTERT ci in primary HFKs. A total of 2359 probes demonstrated altered mRNA expression in hTERT wt HFKs compared to 5467 changes by hTERT ci D868A (Figure 1B). Interestingly, 2077 of the 2359 (88%) probes altered by hTERTwt were also altered by hTERT ci D868A. These gene expression alterations seen following hTERT expression, therefore, are largely independent of the reverse transcriptase function. In all, 1258 changes were shared by hTERT wt, hTERT ci D868A, and E6, requiring significant additional considerations to focus our study (Figure 1C). AIB1 was present in these shared genes altered by hTERT independent of its catalytic function (Figure 1C, D).

### 3.2 HPV‐16 E6 increases *AIB1* mRNA and AIB1 protein

**Figure 2:**
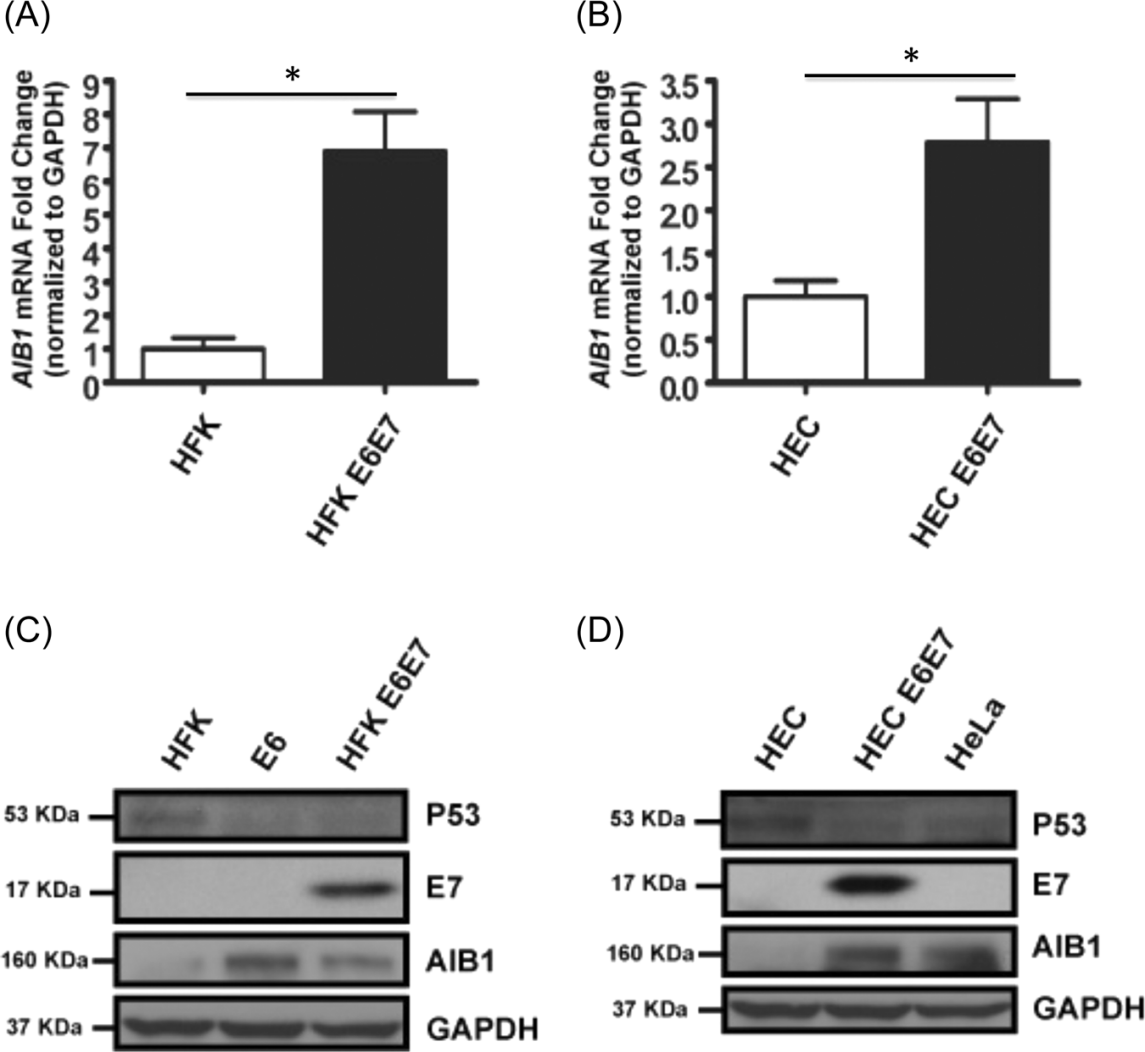
AIB1 mRNA and AIB1 protein are increased in multiple cell types immortalized by HPV‐16 E6/E7. Primary HFKs and primary human ectocervical cells (HECs) were transduced with pLXSN‐based retroviruses with HPV‐16 E6/E7 and selected with G418 as previously described. Quantitative RT‐PCR was performed with gene‐specific primers for *AIB1* for (A) HFKs with and without E6/E7 expression and (B) HECs with and without E6/E7 expression, normalized to GAPDH. *n* = 3. Bars represent mean ± SD. *p < 0.05. AIB1 protein levels were quantified by Western blot in (C) HFKs with and without E6/E7 expression and (D) HECs with and without E6/E7 expression and HeLa cells (HPV18‐positive cervical cancer cell line). Lysates were separated by 4%−20% gradient SDS‐PAGE. Antibodies were used to detect AIB1 (1:1000, 5E11, Cell Signaling), P53 (1:1000, Pab 1801, Santa Cruz), and HPV16‐E7 (1:1000, ED17, Santa Cruz) and GAPDH (1:2000, FL‐335, Santa Cruz) served as a loading control.

Using the same cell lines above, we verified that E6 expression led to increases in *AIB1* transcript levels by real‐time RT‐PCR compared to empty vector alone (Figure 1E). AIB1 protein levels were assessed by Western blot analysis and shown to be increased by E6 compared to the empty vector alone (Figure 2C, middle lane), suggesting a novel cellular target of HPV E6.

### 3.3 *AIB1* mRNA and AIB1 protein are increased in multiple cell types immortalized by HPV‐16 E6/E7

Then we investigated whether *AIB1* mRNA and AIB1 protein levels were increased in HPV‐immortalized HFKs. We transduced HFKs with retrovirus LXSN‐HPV‐16 E6/E7 and grew cells beyond their normal proliferative stage then isolated protein and RNA. *AIB1* mRNA levels were tested by quantitative real‐time RT‐PCR using gene‐specific primers. As expected, *AIB1* mRNA was increased in HFKs (Figure 2A) immortalized by HPV‐16 E6/E7. AIB1 protein was increased in HFKs immortalized by E6/E7 as shown by Western blot analysis using the anti‐AIB1 antibody (Figure 2C). Anti‐P53 (Pab 1801; Santa Cruz) was used to indirectly verify E6 expression while HPV16‐E7 expression was measured directly by anti‐E7 antibody (ED17; Santa Cruz). GAPDH served as a loading control (Sigma).

We next examined whether the increase in AIB1 is restricted to the single HFK cell type, or if AIB1 increases occur in other cell types. As with the HFKs, primary human ectocervical cells (HECs) were infected with LXSN‐HPV‐16 E6/E7 and cells were grown beyond their normal proliferative. Expression of E6/E7 in HECs is known to lead to immortalization. AIB1 protein levels were measured by Western blot analysis and *AIB1* mRNA by quantitative real‐time PCR. Consistent with our data in HFKs, we observed increases in both *AIB1* mRNA and protein in the immortalized E6/E7‐expressing HECs compared to uninfected HECs (Figure 2B, D).

### 3.4 Low‐risk HPV E6 does not induce *AIB1* mRNA in keratinocytes

**Figure 3:**
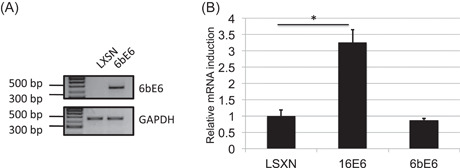
Low‐risk HPV E6 does not induce expression of AIB1 in genital keratinocytes. HFKs were infected with retroviruses of pLXSN vector or HPV 6b E6 (low‐risk type) and AIB1 mRNAs were measured with real‐time RT‐PCR. (A) Confirmation of expression of HPV 6b E6 by RT‐PCR. (B) Quantitative RT‐PCR for detection of AIB1 mRNAs in triplicate. *n*  =  3. Bars represent mean ± SD. *p < 0.05.

**Figure 4:**
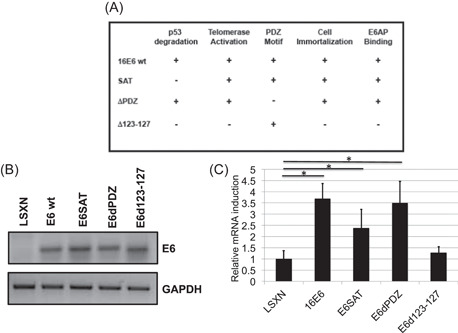
Expression of AIB1 in genital keratinocytes correlates with ability of E6 to induce telomerase and p53 degradation. HFKs were infected with retroviruses of pLXSN vector or HPV16E6 mutants. (A) The HPV‐16 E6 protein is 151 aa in length, and has several cellular targets or functions in the host cells. Three mutations were introduced: SAT, a mutated protein that substitutes the HPV‐16E6 residues 8−10 with the “low‐risk” HPV‐6b E6 residues; truncated E6 protein ΔPDZ, which is a loss of residues 141−151; E6 with deleted residues Δ123−127. These E6 mutants are summarized the altered functions of these mutated proteins. (B) Confirmation of expression of HPV16 E6 by RT‐PCR. (C) Quantitative RT‐PCR for detection of AIB1 mRNAs. Assays were triplicate and bar graphs were shown as mean ± SD, * *p* < 0.05.

HFKs were transduced with pLXSN‐based retroviruses with empty vector, high‐risk HPV‐16 E6, and low‐risk HPV‐6b E6 and selected with G418 as previously described. As before, *AIB1* mRNA levels were evaluated by quantitative RT‐PCR using gene‐specific primers. *AIB1* mRNA level was increased by high‐risk HPV‐16 E6 (Figure 3B). However, *AIB1* levels remained unchanged in cells transduced with the low‐risk HPV‐6b E6 as in control cells (Figure 3B). E6 expression from high‐ and low‐risk types was confirmed by RT‐PCR as shown in Figures 3A and 4B. We next investigated if in vitro activation of the AIB1 reporter was specific to the high‐risk HPV‐16 or if AIB1 could be activated by the low‐risk HPV‐6b type. A 1.6 kb AIB1 promoter construct incorporates the −1250 to +350 region of exon 1 of AIB1 with the pGL3 backbone, utilizing the luciferase reporter system. The AIB1 promoter construct was transiently cotransfected in HFKs as before with the pRL‐CMV plasmid (Promega) and now with expression vectors pJS55‐empty vector, pJS55‐HPV‐16 E6, or pJS55‐HPV‐6b E6. Cells were lysed 24 h after transfection. Luciferase activity was measured as described in Section 2. Based on these results, the 1.6 kb AIB1 promoter is activated by both high‐risk HPV‐16 E6 and low‐risk HPV‐6b E6 by more than twofold (Figure S3A). In this case, we observed inconsistent results from induction of AIB1 mRNA and in vitro reporter activity by low‐risk E6. Reporter assay is an important tool in analyzing *cis* and *trans* factors, and the results need to be interpreted carefully.^87^ This inconsistency maybe because the construct does not have all the promoter elements in it for full effects of E6 on activity, so the E6 effect may be indirect.

**Figure 5:**
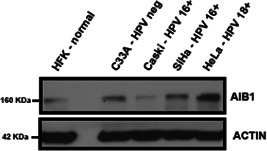
AIB1 is differentially expressed in cervical cancer cell lines compared to normal genital keratinocytes. Lysates were harvested from C33A, Caski, SiHa, and HeLa cervical cancer lines. AIB1 protein levels were quantified by Western blot. HFKs served as a normal control. Lysates were separated by 4%−20% gradient SDS‐PAGE. Antibodies were used to detect AIB1 (1:1000, 5E11, Cell Signaling) and Actin (1:5000, Sigma) served as a loading control.

### 3.5 Induction of AIB1 correlates with the ability of E6 to immortalize keratinocytes with E7

To further probe E6 activity on AIB1 induction, we introduced mutations to the E6 protein (Figure 4A). The SAT substitution mutant replaces amino acids 8−10 from high‐risk HPV‐16 with the amino acids SAT found in the low‐risk HPV‐6b amino acid sequence. The SAT mutant has been previously shown to retain its ability to immortalize human mammary epithelial cells (HMECs) while losing its ability to degrade p53.^40^ For the SAT mutant, the PDZ motif, ability to activate telomerase, and E6AP binding remains intact (Figure 4A). A second mutant, the ΔPDZ, represents a truncation of the last 10 amino acids, causing loss of its C‐terminal PDZ binding motif. Immortalization of HFKs in combination with 16E7, E6AP binding, telomerase activation, and p53 degradation functions are intact (Figure 4A). Conversely, the Δ123−127 retains only PDZ binding capabilities. This deletion mutant lacks the ability to induce telomerase, degrade p53, bind E6AP, and cannot immortalize HFKs in combination with E7. These mutant constructs were previously published.^40^ We confirmed the expression of E6 by RT‐PCR (Figure 4B). E6ΔPDZ demonstrated similar activity compared to wt E6, suggesting that the PDZ motif is not required for AIB1 induction. E6SAT (defective for p53 degradation but active for telomerase induction and cell immortalization) is partially defective for AIB1 induction while the E6Δ123−127 (defective for p53 degradation, telomerase activation, and cell immortalization) is also defective for AIB1 induction. These data suggested a correlation of E6 induction of AIB1, telomerase, and p53 degradation.

This AIB1 reporter plasmid was transiently cotransfected in HFKs with the expression vectors pJS55‐empty vector or pJS55‐HPV‐16 E6 expressing wild‐type or mutant E6. Cells were also cotransfected with the pRL‐CMV plasmid (Promega), which contains the *Renilla reniformis* luciferase gene as a transfection control. Twenty‐four hours after transfection, cells were lysed and luciferase activity was measured. Based on these results, the reporter assay showed that the AIB1 promoter is activated by HPV‐16 E6 wt more than twofold (Figure S3B). However, AIB1 reporter activity was unexpectedly diminished by E6 mutants. Again, these inconsistencies may be due to the length of the AIB1 promoter and other indirect effects by E6.

### 3.6 AIB1 is increased in cervical cancer cell lines

Given our previous study relating AIB1 and E6AP^74^ as well as publications showing AIB1‐interacting proteins are targets of HPV,^66^, ^84^ we screened multiple cervical cancer cell lines for AIB1 expression. Western blot analysis using anti‐AIB1 antibody was performed to investigate AIB1 protein levels in SiHa (HPV‐16 positive), Caski (HPV‐16 positive), HeLa (HPV‐18 positive), and C33A (HPV negative) cell lines and normal HFK as a control. An increase in AIB1 protein could be seen in three of the four cell lines compared to normal (Figure 1).

### 3.7 AIB1 is a biomarker for cervical dysplasia and carcinoma

**Figure 6:**
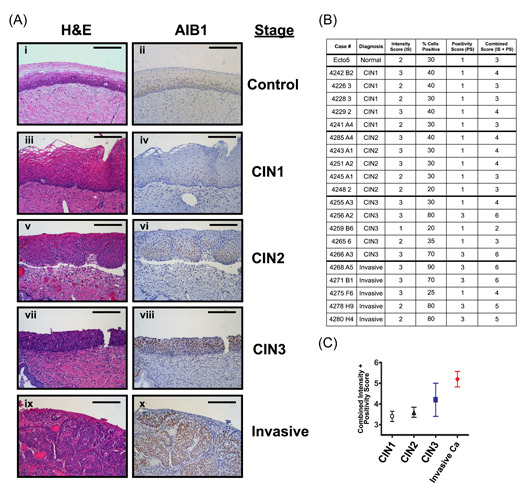
AIB1 protein expression positively correlates with disease stage in cervical dysplasia and neoplasia in vivo. (A) Tissues of cervical intraepithelial neoplasia stage 1 (CIN1), CIN2, CIN3 or carcinoma in situ, and invasive cervical carcinoma were acquired. Immunohistochemical staining with hematoxylin and eosin (iii, v, vii, ix) and for AIB1 protein (iv, vi, viii, x) was performed. Representative images are shown. Relevant controls are shown, staining with hematoxylin and eosin (i) and for AIB1 protein (ii). Scale bar = 50 µm. (B) To quantify AIB1 expression, stained slides were subjected to a randomized, blinded review by a board‐certified clinical pathologist. A subset of slides was scored multiple times to demonstrate reproducibility. For each sample, the case number and diagnosis is provided with the corresponding intensity score, the percentage of positive cells, the corresponding positivity score, and the combined score. Each case received an intensity score from 0 to 3 (0 = *negative*, 1 = *weak*, 2 = *moderate*, 3 = *intense*) and the percentage of positive cells was recorded, which was converted to a positivity score (0 = less than 10%, 1 = 11%−40%, 2 = 41%−69%, 3 = 70%−100%). Combined scores were calculated by adding the intensity score and positivity scores. (C) Mean and standard deviation of combined scores are shown.

Given the increased expression of AIB1 protein in cervical cancer cell lines, we investigated the levels of AIB1 during initiation and/or progression of neoplasia in vivo by obtaining clinical samples to perform immunohistochemistry. Twenty‐one cervical tissues were acquired which represented different pathological stages –one normal tissue core and five tissue cores for each of the following pathological stages: Cervical Intraepithelial Neoplasia Stage 1 (CIN1), CIN2, CIN3 or carcinoma in situ, and invasive cervical carcinoma. Following pathological review, immunohistochemical staining with hematoxylin and eosin (Figure 6A **‐**i, iii, v, vii, ix) and for AIB1 protein (Figure 6A **‐**ii, iv, vi, viii, x) was performed. Strikingly, AIB1 showed positivity in the nucleus of cells in the immediate suprabasal epithelium while nuclei of the basal epithelium stained negative, most clearly seen in the CIN1 sample (Figure 6A, iv). As clinical stage increases from normal to CIN1, 2, CIN3 carcinoma in situ and invasive carcinoma (Figure 6A‐ii, iv, vi, viii, x), AIB1 staining is increased along the epithelial layers, with more cells staining positively as stage increased. Also, for cases of invasive cervical carcinoma, AIB1 staining was specific to cancerous lesions (Figure 6A, x).

To quantify AIB1 expression, stained slides were subjected to a randomized, blinded review by a board‐certified clinical pathologist. A subset of slides was scored multiple times to demonstrate reproducibility. Each case received an intensity score from 0 to 3 (0 = *negative*, 1 = *weak*, 2 = *moderate*, 3 = *intense*) and the percentage of positive cells was recorded, which was converted to a positivity score (0 = less than 10%, 1 = 11%−40%, 2 = 41%−69%, 3 = 70%−100%). Combined scores were calculated by adding the intensity score and positivity scores (Figure 6B). Mean and standard deviation of combined scores are shown (Figure 6C). An overall trend in increased positive staining can be seen in the images. Invasive carcinoma showed combined scores that are significantly higher than both CIN1 and CIN2 (*p* = 0.0012 vs. CIN1; *p* = 0.0130 vs. CIN2) but not CIN3 (*p* = 0.2903; *p* values as calculated by Student's *t* test). The increased AIB1 staining seen in invasive carcinoma was statistically significant from CIN1 and CIN2 but not CIN3 (*p* = 0.0012 vs. CIN1; *p* = 0.0130 vs. CIN2; as calculated by Student's *t* test). Given these data, AIB1 may serve as a biomarker for the progression of cervical cancers and offers an avenue for potential therapeutic development.

### 3.8 AIB1 is also increased in mouse cervical dysplasia and carcinoma induced by HPVE6E7 and estrogen

**Figure 7:**
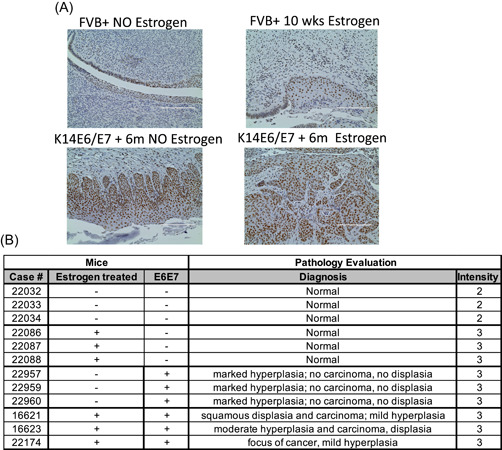
AIB1 protein expression in mouse cervical lesion and cancer models induced by HPV16 E6E7 and estrogen. The murine models (FVB and C57BL/6) of human cervical carcinogenesis using the human Keratin14 (hK14) promoter‐driven HPV16 E6E7 oncogenes and estrogen treatment were generated in Lambert laboratory (A). FVB control mice and estrogen‐treated mice as shown in upper panels (A), respectively. HPV16 E6E7 transduced mice develop CIN lesion as shown in left lower panel and mice with E6E7 and estrogen develop invasive carcinoma as right lower panel (A). AIB1 protein was stained with IHC as described in Section 2. Pathology evaluation was shown in panel B.

Lambert and colleagues genetically developed the murine models (FVB and C57BL/6) of human cervical carcinogenesis using the human Keratin 14 (hK14) promoter‐driven HPV16 E6E7 oncogenes and estrogen treatment.^88^, ^89^, ^90^, ^91^ Untreated HPV16 E6E7 transgenic mice develop CIN lesions and estrogen‐treated transgenic mice develop high‐grade dysplasias and squamous cell carcinoma. These preclinical models are useful for the basic biology of HPV oncogenes in vivo and the development of prevention and treatment. Immunohistochemistry was performed on mouse tissue and AIB1 expression was found to be increased in mouse cervical neoplasia and invasive carcinoma (Figure 7A). Samples were reviewed randomly, blinded, and reproducibly scored by a board‐certified pathologist. AIB1 was expressed in the nucleus in epithelium. AIB1 staining was scored from 0 to 3: 0 for negative; 1 for weak; 2 for moderate; 3 for intense. The results showed that AIB1 intensity was higher in both high‐grade dysplasias (E6E7) and squamous cell carcinoma (E6E7 plus estrogen treatment) than in normal controls (Figure 7B). Interestingly, the percentage of stained cells in estrogen‐treated mice was lower than that in either high‐grade dysplasias or squamous cell carcinoma, while intensity was similar (Figure 7B). This indicated that estrogen treatment may also regulate the expression of AIB1. Thus, our data demonstrate that AIB1 may serve as a new biomarker in cell models, mouse models, and human cervical precancerous lesions and invasive carcinoma.

### 3.9 Knockdown of AIB1 in HPV E6E7 immortalized cells decreased cell proliferation

**Figure 8:**
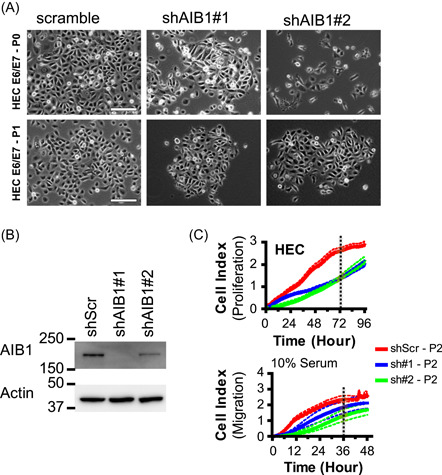
Knockdown of AIB1 decreases cell proliferation and migration HPV16 E6E7 immortalized HEC. Cells were seeded at 5 × 10^5^ cell in dish and allowed to form a monolayer overnight. Lentiviruses with either control or AIB1 shRNAs infected cells with polybrene in complete media for 2 days. Puromycin selection was carried for 3 days. Five days post infection (upper panel A), the cells were trypsinized and replated in dishes for 5 days (lower panel A), and cell lysates were harvested for western blot (B). Cell proliferation arrays were set up in 16‐well E‐Plate VIEW (ACEA #AE06324738001) and growth rate/proliferation and migration were recorded as cell index by real‐time monitoring using an electric cell‐substrate impedance sensor system (ECIS) over 4 days. Proliferation and migration assays suggested that shRNA against AIB1 significantly decreased cell proliferation with growth curves in upper panel (C), and cell migration in lower panel (C). All experiments were set up in triplicate (three wells for each), one dot line represented cell proliferation or migration curve from one well. The values from triplicated wells with proliferation assay (at 72 h) and migration assay (at 36 h) as black dot lined were analyzed, differences between control and shRNA groups were statistically significant (*p* < 0.05).

To further study the biological role of AIB1 in cervical cancer, we introduced interfering short hairpin RNA sequences in E6E7 immortalized cervical epithelial cells (HEC). As shown in Figure 8B, shRNA against AIB1 significantly decreased expression of AIB1 proteins detected by Western blot in E6E7 immortalized HEC 5 days after lentiviral infections. Cell proliferation and migration arrays were set up in 16‐well E‐Plate VIEW (ACEA #AE06324738001) and growth rate/proliferation was recorded as cell index by real‐time monitoring using an ECIS over 4 days. Proliferation and migration assays suggested that shRNA against AIB1 significantly decreased cell proliferation (upper panel) and migration (lower panel) with the growth curves of Figure 8C. Thus, our data demonstrated an essential role of AIB1 in the cell proliferation of HPV E6E7 immortalized cervical cells.

## 4 DISCUSSION

Our array data identified a gene set of 1258 changes that were altered in HFKs expressing E6, hTERT wt, and hTERT ci D868A. AIB1 drew our interest, given data demonstrating E6AP can target AIB1 for degradation^74^ as well as recent publications showing AIB1‐interacting proteins to be targets of HPV.^66^, ^68^, ^76^, ^83^, ^84^ We validated that E6 increases *AIB1* mRNA and AIB1 protein (Figures 1 and 2). We screened multiple cervical cancer cell lines for AIB1 expression and found it to be increased compared to normal HFKs (Figure 5). AIB1 protein levels were also increased in HPV‐immortalized cell lines and cervical cancer cell lines (Figure 2). Increased *AIB1* mRNA levels appear to be specific to the high‐risk HPV‐16 versus the low‐risk HPV‐6b E6 proteins (Figure 3). E6 mutagenesis demonstrated that induction of AIB1 by HPV16 E6 correlated with its ability to induce cell immortalization together with E7.

Interestingly, an early study from Riegel's laboratory demonstrated that nuclear export is required for proteasomal degradation of AIB1 and involved E6AP.^74^ Knockdown of E6AP prevents proteasomal degradation of AIB1, while overexpression of E6AP promotes AIB1 degradation. Either pretranslocation or posttranslocation into the nucleus, AIB1 forms a complex either directly through its COOH terminus with E6AP or with an intermediary E6AP‐regulated protein. Ultimately, an E6AP‐AIB1 complex potentiates transcription in the nucleus.^74^ Since the loss of E6AP binding diminishes E6 physical interactions with its targets in host cells,^51^, ^92^, ^93^, ^94^, ^95^ it is possible that E6AP may be a mediator of AIB1 functions in cervical cancer by E6.

E6 proteins from HR HPVs increase cellular telomerase activity via both transcriptional and posttranscriptional mechanisms^47^, ^52^, ^96^, ^97^, ^98^, ^99^, ^100^, ^101^, ^102^, ^103^, ^104^, ^105^, ^106^ and this activation is considered a critical and rate‐limiting step in the progression to malignancy. For example, a study at Burk's laboratory supports the notion that activation of the hTERT promoter by E6 genes is uniquely associated with oncogenic types, independent of evolutionary relationships.^107^ New evidence is emerging for the noncanonical role of telomerase in tumor progression including invasion and resistance to therapeutic drugs.^102^ We described noncanonical functions of TERT several years ago,^42^ including the induction of cell gene expression, and decided to explore whether TERT/TERT may play a role in tumor progression beyond its function in cell immortalization. For example, mTERT has been ascribed roles in altering apoptotic responses,^108^, ^109^, ^110^ tumor formation in mice,^111^, ^112^ stem cell migration and renewal,^113^, ^114^ and chromatin remodeling.^115^ Artandi's laboratory at Stanford has shown that mTERT can not only augment breast cancer development in mice but that it regulates the transcription of genes responsive to the Wnt/beta catenin pathway.^113^, ^114^ TERT also exhibits antiapoptotic activity and this activity is independent of telomere‐elongation and telomerase activities.^108^, ^116^, ^117^ More recently, a splice‐variant of hTERT (Δ4−13) was discovered that contains an in‐frame deletion of exons 4 through 13 that encodes the catalytic domain of telomerase.^71^ Overexpression of the Δ4−13 splice‐variant significantly elevates the proliferation rate of several cell types without enhancing telomerase activity. Our previous findings are the first to link hTERT noncatalytic activity to cell immortalization, which was previously believed to be solely dependent upon hTERT RT activity.^42^ These activities of hTERT involve the expression of BMI1 induced by E6 independently of telomere lengthening. Here, we showed that AIB1 is a novel target for E6 in keratinocytes and cervical cells. Interestingly, hTERT and enzymatic inactive hTERT also induced expression of AIB1, suggesting possibilities that E6 associated functions in host cells via noncanonical functions of hTERT proteins.

Reporter assays have proven to be an important tool in analyzing *cis* and *trans* factors that influence gene expression.^87^ We noted the inconsistency of AIB1 induction by low‐risk HPV E6 and HPV16 E6 mutants using endogenous mRNA by RT‐PCR and luciferase activity by reporter assays in this study (Figures 3, 4, and S3). These findings might be due to several possibilities: 1. This reporter construct does not have all the promoter elements in it for full effects of E6 on activity; 2. the E6 effect may be indirect; 3. E6 induces AIB1 through more than one mechanism at multiple levels simultaneously.

Tissue sections of CIN1, CIN2, and CIN3 and invasive carcinoma allowed us to measure AIB protein levels in vivo. Unlike the stem cell renewal protein BMI1, AIB1 expression was negative in the basal progenitor cells. Suprabasal epithelium stained intensely positive (Figure 6). Intensity and percent positivity scoring revealed a significant increase in AIB1 expression in invasive lesions compared to CIN1, CIN2, and CIN3 (Figure 6). Indeed, AIB1 has been implicated in cancer invasion in a variety of cancer types, including breast, prostate, liver, lung, and others.^69^, ^118^, ^119^, ^120^ A number of studies claim that the mechanism of invasion by AIB1 is through upregulation of matrix metalloproteinases (MMPs).^118^, ^119^, ^120^, ^121^ A very recent study showed that the X protein of the Hepatitis B virus (HBV) stabilizes AIB1 protein, found upregulated in 68% of the hepatocellular carcinoma samples in the study, and correlates with invasiveness. The study also showed the X protein mediates MMP9 activation.^122^ Interestingly, *MMP9* mRNA levels are significantly upregulated in the E6 array data sets (data not shown).

Given the known association of AIB1 expression with invasion, we acquired additional cases of invasive adenocarcinoma and squamous cell carcinoma. Pathological scoring showed no statistical difference between the tissue types. However, clear differences could be distinguished in cellular localization. Whereas AIB expression in squamous cell carcinoma is limited to the nucleus, the AIB expresses in the cytoplasm and cell membrane in adenocarcinoma (Figure S2). Given the well‐characterized function of AIB1 as nuclear receptor coactivator, its nuclear localization in squamous epithelium was not surprising.^69^, ^123^, ^124^ However, studies have described changes in AIB1 cellular localization as well. Mutations in the nuclear localization signal (NLS) lead to a proteasome‐resistant cytoplasmic form of AIB1.^125^ A splice variant, AIB1Δ4, predominantly localizes to the cytoplasm.^126^ Intriguingly, its expression correlates with metastatic potential. Thees data support the potential role of AIB1Δ4 in cervical cancerous lesions in vivo and future studies exploring AIB1Δ4 function in adenocarcinoma of the cervix should be considered.

Lambert lab has shown cooperation of HPV16 E6E7 and estrogen in development of cervical cancer in transgenic models,^88^, ^89^, ^90^, ^91^ suggesting that estrogen is a cofactor in causing cervical cancer in human. It is known that estrogen's carcinogenesis is mediated through a nuclear transcription factor, estrogen receptor α (ERα).^127^ Estrogen is essential not only for the development of cervical cancers in HPV16 transgenic mice but also for the maintenance of those cancers.^128^ Consistent with these findings, Lambert lab demonstrated that ERα antagonists are highly effective at treating cervical cancers and precancerous lesions arising in the HPV16 transgenic mice.^129^, ^130^ We speculate that AIB1 may contribute to this cooperation since AIB1 is an E6 target and serves a cofactor of nuclear receptors including ER.^69^, ^78^, ^131^, ^132^ Thus, AIB1 is not only a E6 target, may also be a target for prevention and therapeutics of human cervical cancer.

Additionally, there was significant variance in AIB1 expression in the invasive cancer cases. In some cases, AIB1 expression levels stained intensely positive (Figures 6 and 7). shRNA assays demonstrated a biological role of AIB1 in the proliferation and migration of HPV immortalized cells (Figure 8). The recent call for AIB1 small molecule inhibitors^133^, ^134^ opens possibilities for personalized medicine approaches where patients are screened by AIB1 expression levels and treated accordingly.

## AUTHOR CONTRIBUTIONS

Jonathan Miller, Aleksandra Dakic, Megan Spurgeon, and Francisco Saenz performed experiments. Bhaskar Kallakury performed pathology evaluation. Bo Zhao, Junran Zhang, Jian Zhu, Qin Ma, and Ying Xu provided tools for analyses. Jonathan Miller drafted the manuscript. Paul Lambert, Richard Schlegel, Anna T. Riegel, and Xuefeng Liu supervised the team during the study and edited the manuscript. All authors read and approved the final version of the manuscript.

## CONFLICTS OF INTEREST

The authors declare no conflicts of interest.

## Supporting information

Supporting information.

Supporting information.

## Data Availability

The data that support the findings of this study are available in the supplementary material of this article and our previous publication (doi:10.1371/journal.ppat.1003284).
