# Supplementary material for: AIB1 is a novel target of the high‐risk HPV E6 protein and a biomarker of cervical cancer progression
Source: J Med Virol. 2022 Apr 27;94(8):3962–77. doi: 10.1002/jmv.27795 (PMC9199254; doi:10.1002/jmv.27795)
Supplement: Supplementary file 1 — Supporting information. [file JMV-94-3962-s002.pdf]

Figure S1

A.

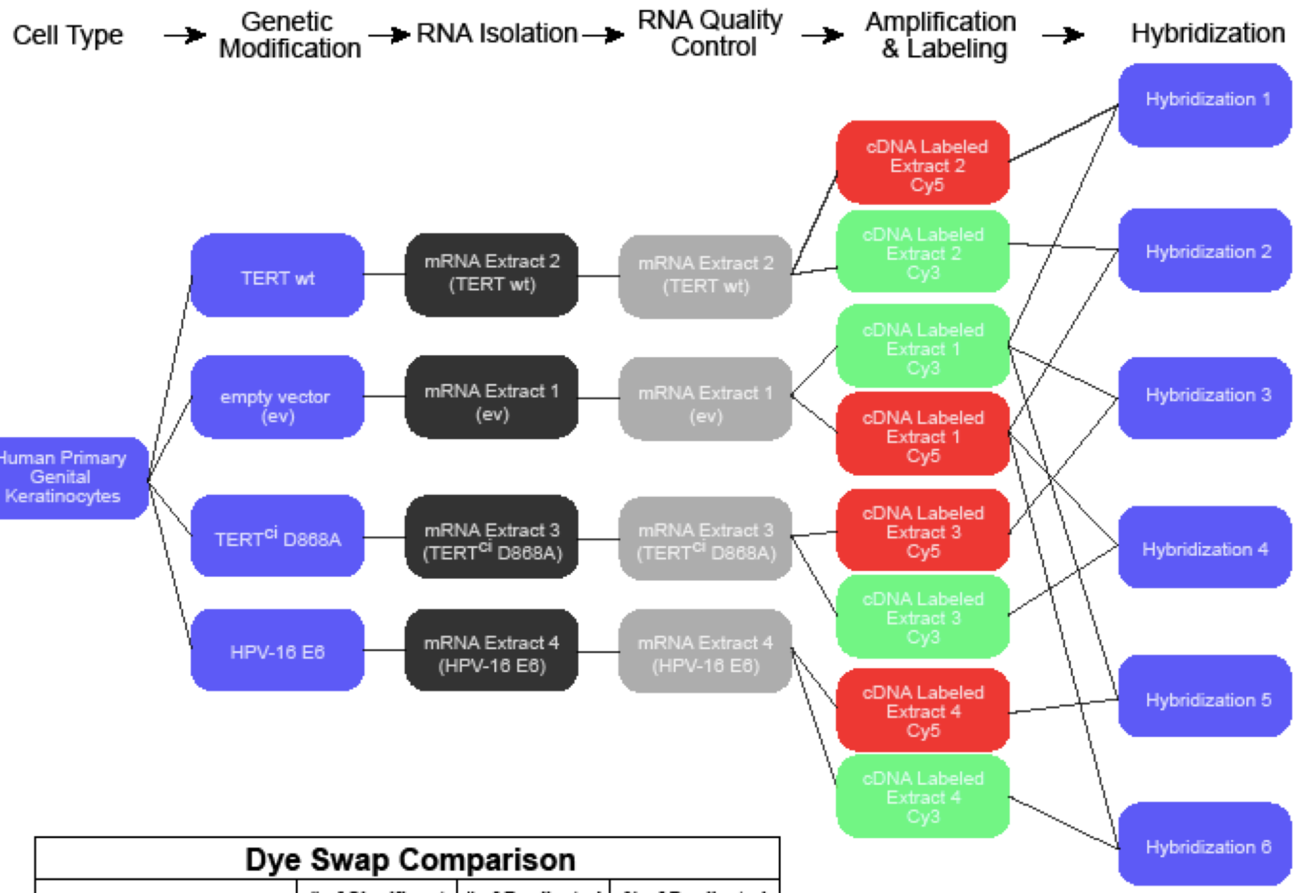

B.

Dye Swap Comparison

Hybridization	# of Significant Changes	# of Duplicated Changes	% of Duplicated Changes
Hybridization 1 (ev vs. TERT wt)	4739	2359	77.0
Hybridization 2 (TERT wt vs. ev)	3062	2359	
Hybridization 3 (ev vs. TERT^{ci} D868A)	7548	5467	83.4
Hybridization 4 (TERT^{ci} D868A vs. ev)	6553	5467	
Hybridization 5 (ev vs. HPV-16 E6)	9055	6991	85.7
Hybridization 6 (HPV-16 E6 vs. ev)	8154	6991	

Figure S2

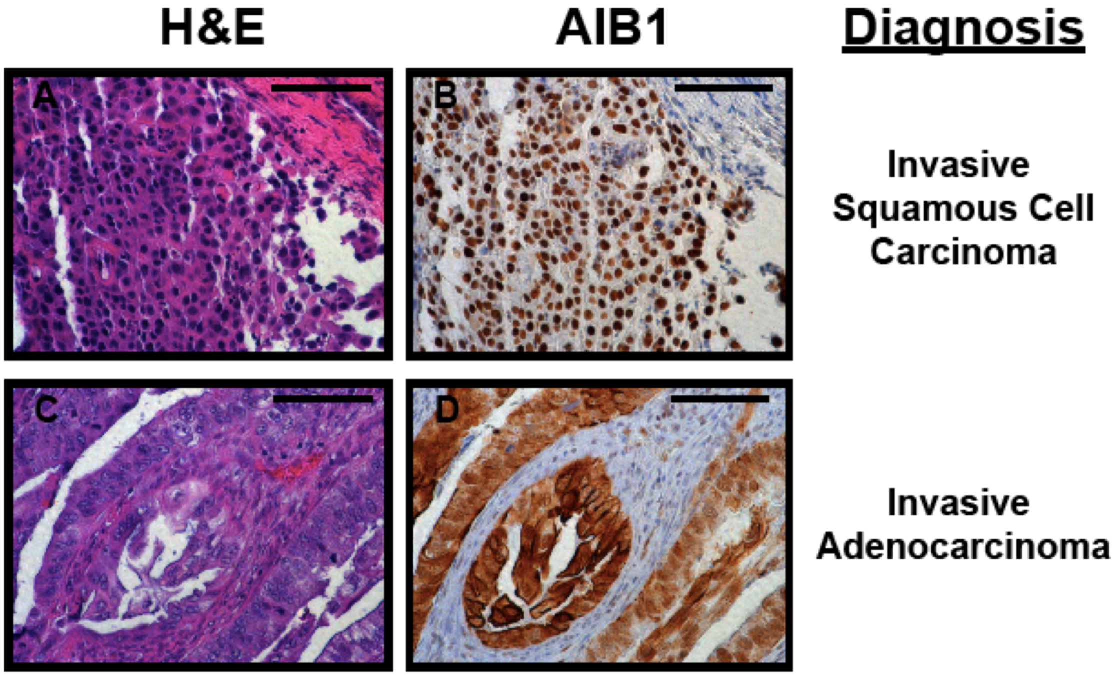
H&E
AIB1
Diagnosis
A
B
C
D
Invasive
Squamous Cell
Carcinoma
Invasive
Adenocarcinoma

Figure S3

A.

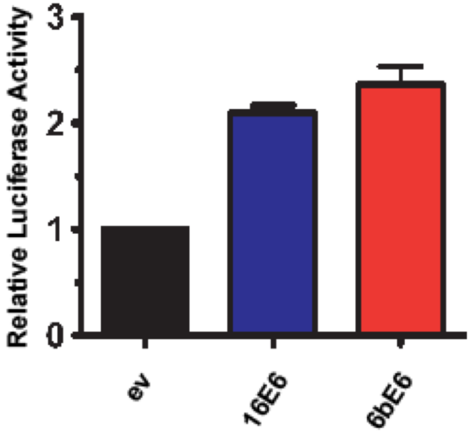
Relative Luciferase Activity
3
2
1
0
ev
16E6
6bE6

B.

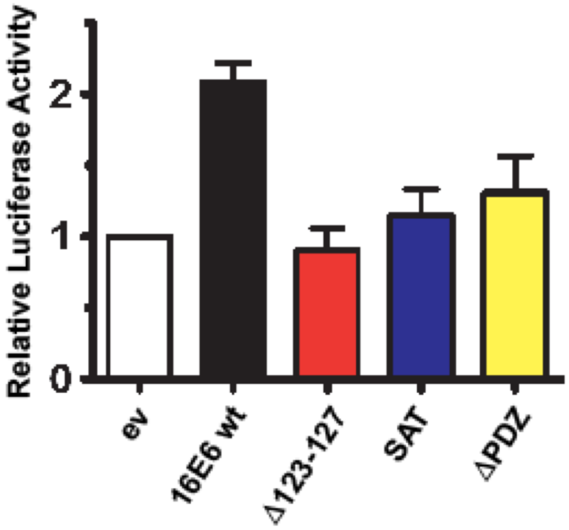
Relative Luciferase Activity
2
1
0
ev
16E6 wt
Δ123-127
SAT
ΔPDZ
