# Supplementary material for: AIB1 is a novel target of the high‐risk HPV E6 protein and a biomarker of cervical cancer progression
Source: J Med Virol. 2022 Apr 27;94(8):3962–77. doi: 10.1002/jmv.27795 (PMC9199254; doi:10.1002/jmv.27795)
Supplement: Supplementary file 2 — Supporting information. [file JMV-94-3962-s001.docx]

**Supplemental Figure Legends**

**Figure S1. Scheme of array-based whole genome expression analysis.** (A) We stably expressed E6, hTERTwt or a catalytically inactive mutated protein hTERTci in primary HFKs. Cells were lysed using the TRIzol reagent and RNA isolated following the manufacturer's protocol (Invitrogen) from samples 12-14 days post-infection. RNA quality was assessed by bioanalyzer. RNA was reverse transcribed and labeled with fluorescent dyes (Cy3 or Cy5) and submitted for array analysis using the Agilent 4 x 44K format. (B) Dye swap comparisons were made and directionally consistent changes were identified. The high percentage of consistency in expression changes are shown in the chart and support the integrity of the data. Changes were called significant if the fold change > 1.33 and p value < 0.01.

**Figure S2. AIB1 protein localizes to different cellular compartments in invasive carcinomas of differing cellular origin.** Tissue from cases of adenocarcinoma and squamous cell carcinoma were acquired as described. Representative images are shown demonstrating the different cellular localization of AIB1 depending on the tissue origin. Tissue staining with hematoxylin and eosin (A, C) and immunohistochemical stain with AIB1 (1:300, 5E11) (B, D) are shown. Scale bar = 100 μm.

**Figure S3. AIB1 reporter is activated by both high-risk and low-risk HPV E6s and diminished by E6 mutants.** (A) HFKs were transiently co-transfected with reporter plasmid pGL3-AIB1-1.6 kb **(86)**, expression vector (vector, HPV-16 E6, or HPV-6b E6) and luciferase control (pRL-delta-CMV). Cells were lysed 24 hrs after transfection and luciferase activity was measured. (B) HFKs were transiently co-transfected with reporter plasmid pGL3-AIB1-1.6 kb, expression vector (HPV-16 E6 wt or Δ123-127, SAT, or ΔPDZ) and luciferase control (pRL-delta-CMV). Cells were lysed 24 hrs after transfection and luciferase activity measured. Protein expression levels of these mutated proteins were not tested. This experiment was repeated three times in triplicate.
